# Clinical epidemiology and outcomes of community acquired infection and sepsis among hospitalized patients in a resource limited setting in Northeast Thailand: A prospective observational study (Ubon-sepsis)

**DOI:** 10.1371/journal.pone.0204509

**Published:** 2018-09-26

**Authors:** Viriya Hantrakun, Ranjani Somayaji, Prapit Teparrukkul, Chaiyaporn Boonsri, Kristina Rudd, Nicholas P. J. Day, T. Eoin West, Direk Limmathurotsakul

**Affiliations:** 1 Mahidol Oxford Tropical Medicine Research Unit, Faculty of Tropical Medicine, Mahidol University, Bangkok, Thailand; 2 Division of Pulmonary, Critical Care, and Sleep Medicine, University of Washington, Seattle, United States; 3 Sunpasitthiprasong Hospital, Ubon Ratchathani, Thailand; 4 Centre for Tropical Medicine and Global Health, Nuffield Department of Medicine, University of Oxford, Churchill Hospital, Oxford, United Kingdom; 5 Department of Tropical Hygiene, Faculty of Tropical Medicine, Mahidol University, Bangkok, Thailand; University of Pittsburgh, UNITED STATES

## Abstract

Infection and sepsis are leading causes of death worldwide but the epidemiology and outcomes are not well understood in resource-limited settings. We conducted a four-year prospective observational study from March 2013 to February 2017 to examine the clinical epidemiology and outcomes of adults admitted with community-acquired infection in a resource-limited tertiary-care hospital in Ubon Ratchathani province, Northeast Thailand. Hospitalized patients with infection and accompanying systemic manifestations of infection within 24 hours of admission were enrolled. Subjects were classified as having sepsis if they had a modified sequential organ failure assessment (SOFA) score ≥2 at enrollment. This study was registered with ClinicalTrials.gov, number NCT02217592. A total of 4,989 patients were analyzed. Of the cohort, 2,659 (53%) were male and the median age was 57 years (range 18–101). Of these, 1,173 (24%) patients presented primarily to the study hospital, 3,524 (71%) were transferred from 25 district hospitals or 8 smaller hospitals in the province, and 292 (6%) were transferred from one of 30 hospitals in other provinces. Three thousand seven hundred and sixteen (74%) patients were classified as having sepsis. Patients with sepsis had an older age distribution and a greater prevalence of comorbidities compared to patients without sepsis. Twenty eight-day mortality was 21% (765/3,716) in sepsis and 4% (54/1,273) in non-sepsis patients (p<0.001). After adjusting for gender, age, and comorbidities, sepsis on admission (adjusted hazard ratio [HR] 3.30; 95% confidence interval [CI] 2.48–4.41, p<0.001), blood culture positive for pathogenic organisms (adjusted HR 2.21; 95% CI 1.89–2.58, p<0.001) and transfer from other hospitals (adjusted HR 2.18; 95% CI 1.69–2.81, p<0.001) were independently associated with mortality. In conclusion, mortality of community-acquired sepsis in Northeast Thailand is considerable and transferred patients with infection are at increased risk of death. To reduce mortality of sepsis in this and other resource-limited setting, facilitating rapid detection of sepsis in all levels of healthcare facilities, establishing guidelines for transfer of sepsis patients, and initiating sepsis care prior to and during transfer may be beneficial.

## Introduction

Sepsis is a syndrome defined by a dysregulated response to infection resulting in significant organ dysfunction and death. Sepsis is a major public health concern. With an aging population, some estimates from the United States (US) and other high-income countries suggest a rising sepsis incidence, albeit with lowered case fatality rates [1–8]. Based on data from seven high-income countries, globally 19 million cases of sepsis (formerly severe sepsis) and 5.3 million deaths were estimated to occur annually [9–11]. Many patients who survive sepsis may incur long-term morbidities [12, 13]. However, these numbers are extrapolated from published population estimates and likely underestimate the true burden of disease, especially in low and middle income countries (LMIC)—which encompass ~87% of the world’s population [10, 14]. Notably, there is a significant paucity of data about the epidemiology and outcomes relating to sepsis in LMIC settings. This is attributable to lack of awareness [15], poor diagnostic classification of sepsis, and resource and cost related issues [16–23]. Even comprehensive disease quantifying initiatives such as the Global Burden of Disease (GBD) 2016 do not currently report sepsis as a cause of death and morbidity [24, 25], hence sepsis could be underrecognized as a health care burden. Given the global threat of sepsis, the World Health Assembly has recently adopted a resolution to improve the approach to sepsis with a specific acknowledgment of LMIC populations [26].

With the scarcity of epidemiology information for community-acquired sepsis in LMICs and that is rarely been systematically investigated in this region, we aimed herein to prospectively evaluate the clinical epidemiology and outcomes of community acquired sepsis in Northeast Thailand.

## Material and methods

### Study design and study site

We conducted a four-year prospective observational study from March 2013 through February 2017 (NCT02217592) to examine the epidemiology and outcomes of individuals with community-acquired infection and accompanying systemic manifestations of infection in a resource-limited setting of Sunpasitthiprasong Hospital in Ubon Ratchathani province, Northeast Thailand. Thailand is an upper-middle income country, spending $264 on health per capita in 2013 [27]. Ubon Ratchathani is the largest province in Northeast Thailand with a population of 1.8 million, covers an area of 16,113 km^2^, and is bordered by Cambodia to the south and Laos to the east. The vast majority of the population of northeastern Thailand reside in rural areas, and upwards of 80% of adults work in agriculture (primarily rice farming). Sunpasitthiprasong Hospital is a public tertiary-care hospital with 1,200 non-ICU beds and 220 ICU beds, and provides care to people living within its catchment area and acts as a referral hospital to 25 district hospitals in the province, smaller hospitals in the main district (Amphoe Muang) of the province, and hospitals in other provinces (particularly, in three adjacent provinces including Amnatcharoen, Sisaket and Yasothorn provinces). Severely ill patients presenting to district and other smaller hospitals are frequently transferred to Sunpasitthiprasong Hospital for its tertiary-care capacities.

### Study participants

We prospectively enrolled adult patients aged 18 years and older who were admitted with a primary diagnosis of suspected or documented infection made by the attending physician. For inclusion, enrollment had to occur within 24 hours of hospital admission, and patients required the presence of at least three systemic manifestations of infection documented in the medical records. The 20 systemic manifestations were consolidated from the 22 variables proposed as diagnostic criteria for sepsis by Surviving Sepsis Campaign (SSC) 2012 (S1 Table) [28]. This definition preceded the subsequent requirement for organ failure in the more recent definition of sepsis used in this paper. We excluded patients who were suspected of having hospital-acquired infections determined by the attending physician, were hospitalized within 30 days prior to the current admission, or were transferred from other hospitals with a total duration of hospitalization >72 hours.

The study team of trained research nurses sequentially screened all medical patients by conducting ward rounds and reviewing admission logs in the emergency department, medical wards and medical ICUs twice daily (morning and afternoon) on each working day. Nurses in the emergency department also notified the study team directly about potentially eligible patients. Written, informed permission was obtained from participants prior to enrollment. For illiterate participants, the study information was read to the participant and their impartial witness, then fingerprinted and signed informed consent was obtained from the participant and their representative, respectively, before enrollment.

### Study team point-of-care assessments

Following enrollment, patients were evaluated by the study nurses at the bedside using four point-of-care assessments: a whole blood lactate Rapid Diagnostic test (RDT) (Lactate Pro 2, Arkray Global Business Inc., Australia), a whole blood glucose RDT (ACCU-CHECK Performa, Roche Diagnostic, Germany), pulse oximetry (Nellcor N-65, Covidien plc., Ireland) and the Glasgow Coma Scale (GCS). Blood samples (4–10 mL) were also collected for culture using BD BACTEC automated blood culture system (Becton-Dickinson, Sparks, MD, USA). The results were reported to the attending physicians. The study did not involve any clinical interventions and all medical treatment was provided by the attending physicians and respective medical teams.

### Data collection

A case report form (CRF) was developed and validated for use in the study and encompassed demographic, clinical and laboratory data during transfer and on admission and enrollment. Data were collected from the medical charts as well as the hospital and microbiology databases. Twenty eight-day mortality data were collected via telephone contact if subjects were no longer hospitalized and had been discharged alive.

We conducted the study in full compliance with the principles of good clinical practice (GCP), and the ethical principles of the Declaration of Helsinki. The study protocol and related documents were approved by Sunpasitthiprasong Hospital Ethics Committee (039/2556), the Ethics Committee of the Faculty of Tropical Medicine, Mahidol University (MUTM2012-024-01), the University of Washington Institutional Review Board (42988) and the Oxford Tropical Research Ethics Committee at the University of Oxford (OXTREC172-12).

### Definitions

Sepsis was defined as an infection with organ dysfunction in accordance with the 2016 International Consensus (Sepsis-3) guidelines for sepsis [29, 30]. A modified SOFA score ≥2 was used to define organ dysfunction and was calculated as the sum of respiratory, coagulation, liver, cardiovascular, central nervous system, and renal parameters +/-24 hours of screening [29, 30]. The study was initiated in 2012 prior to the Sepsis-3 definition [29, 30], and inotropic and vasopressor agent doses were not recorded into the CRF. For the cardiovascular component of the SOFA score, the scoring was modified such that subjects were scored a maximum of 2 (on a 4-point scale) if they received only dobutamine or dopamine, and scored a maximum of 3 if they received epinephrine or norepinephrine. For the respiratory component of the SOFA score, as PaO_2_/FiO_2_ indices were not available for the majority of subjects due to infrequency of arterial blood gas tests, the score was modified as follows: Subjects were scored a maximum of 2 (4-point scale) if they received advanced respiratory support (endotracheal tube, gas powered or electrical powered mechanical ventilation) and arterial blood gas test was not performed. For patients who required mechanical ventilation, the GCS verbal score was calculated by the following formula: (-0.3756) + GCS Motor*(0.5713) + GCS Eye*(0.4233) [31].

Blood culture result from blood samples collected within 24 hours of admission was evaluated. Because of the difficulty in establishing their clinical significance, organisms frequently associated with contamination including coagulase-negative staphylococci, alpha-haemolytic streptococci, *Micrococcus* spp, *Diptheroid* spp., *Propionibacterium* spp, *Corynebacterium* spp, or *Bacillus* spp were excluded from the analysis.

Presenting clinical syndromes were classified based on the primary diagnoses of attending physicians. The clinical syndromes were grouped into acute febrile illness, lower respiratory infection, diarrheal illness, sepsis, septic shock and others. Acute febrile illness included the primary diagnosis of systemic infection noted by attending physicians. Lower respiratory infection included the primary diagnosis of bronchitis, infected bronchiectasis and pneumonia. Diarrheal illness included the primary diagnosis of acute gastroenteritis.

### Statistical analysis

Data were summarized with medians and interquartile ranges (IQR) for continuous measures, and proportions for discrete measures. IQRs are presented in terms of 25^th^ and 75^th^ percentiles. Continuous variables and proportions were compared between groups using Mann-Whitney U tests and Chi-square tests, respectively.

The primary outcome was 28-day mortality and was determined for the overall cohort as well as for subjects classified as sepsis and non-sepsis. Secondary outcomes included time to death, and length of stay in hospital. We performed survival analyses using the Kaplan-Meier method and Cox proportional hazard models for mortality outcome. Time was measured from the admission date. The hazard ratio for 28-day mortality was also assessed separately by each modified SOFA component. For this analysis, organ dysfunction was deemed to be present if the modified SOFA score was ≥1 for each system. The multivariable models were developed using purposeful selection. All analyses were performed with STATA 14.2 (StataCorp, College Station, TX, USA). The final database with the data dictionary are publicly available online (https://dx.doi.org/10.6084/m9.figshare.5544592).

## Results

### Patient population

**Fig 1:**
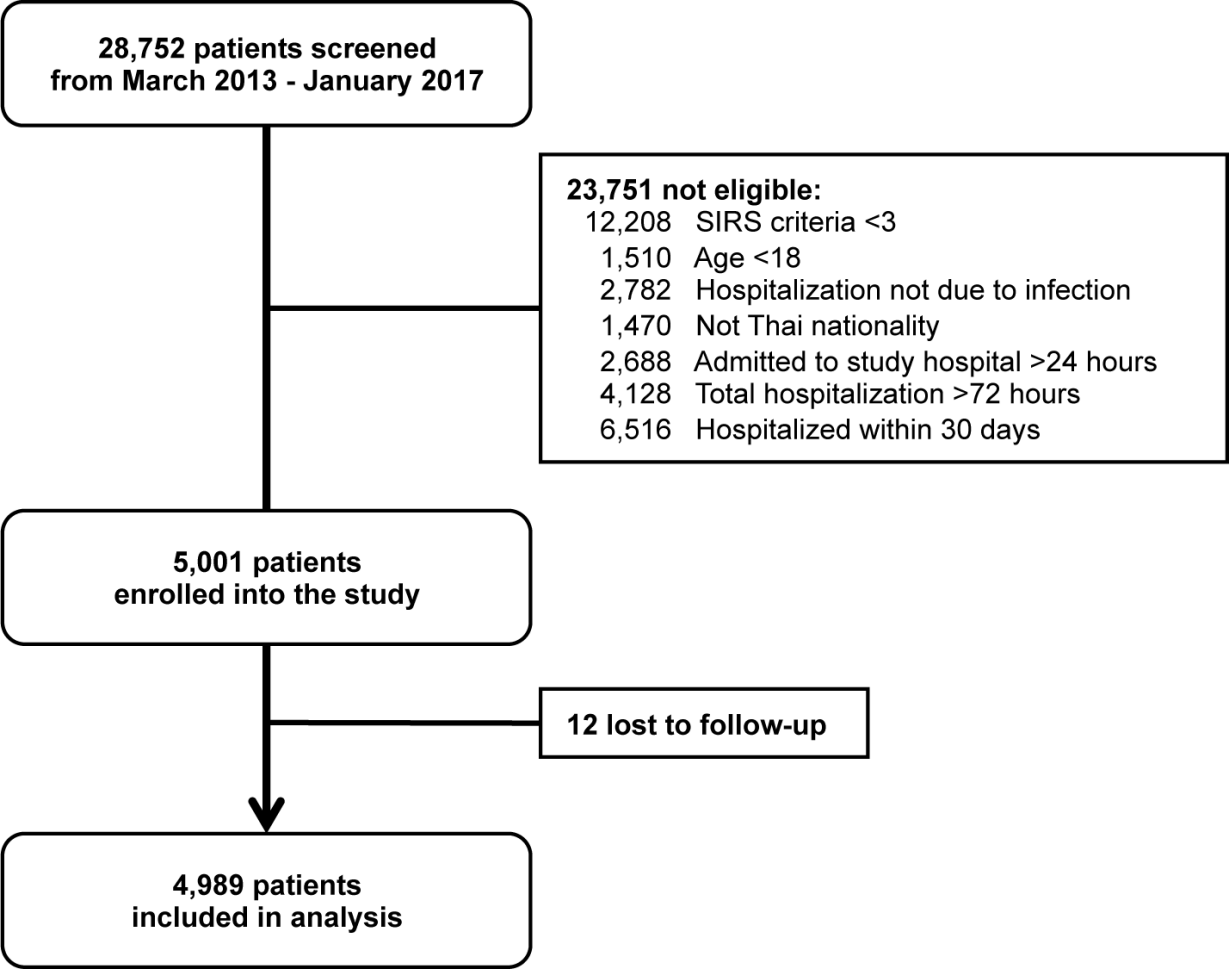
Study flow diagram.

Over the 4 year study period, 28,752 patients admitted to Sunpasitthiprasong Hospital were screened by the study team (Fig 1). The most common exclusion criteria were: having fewer than three systemic manifestations of infection (12,208; 42%), being hospitalized in the past 30 days (6,516; 23%), and transferring from other hospitals with a total duration of hospitalization more than 72 hours (4,128; 14%). A total of 5,001 patients were enrolled into our study, and 12 patients (0.2%) were excluded from the analysis due to unknown 28-day mortality outcome.

**Fig 2:**
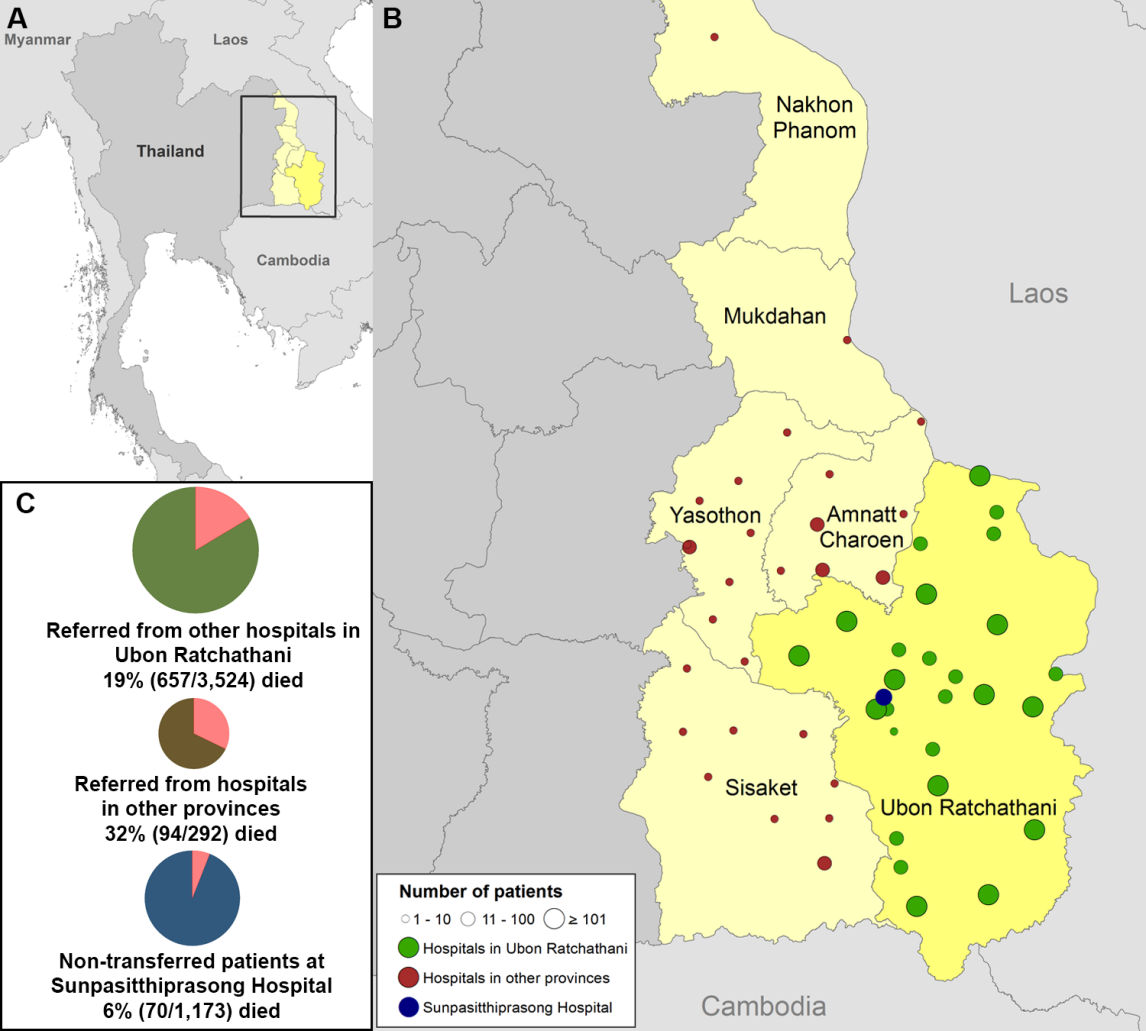
Geographical distribution of the referring hospitals, and 28-day mortality of non-transferred and transferred patients. (A) Map of Thailand. Yellow areas represent provinces from which patients were transferred. (B) Locations of hospitals. Navy blue circle represents the study hospital, Sunpasitthiprasong Hospital. There were a total of 63 referring hospitals; 33 were located in Ubon Ratchathani province, 25 were located in the three adjacent provinces, and 5 were located in the other provinces. Green circles represent 33 referring hospitals located in Ubon Ratchathani province (7 were in Mueang district). Brown circles represent referring hospitals located in three adjacent provinces and the other provinces. (C) Three pie charts represent 28-day mortality. The navy blue, green and brown pie charts represent non-transferred patients, patients transferred from other hospitals in Ubon Ratchathani, and patients transferred from other provinces, respectively. ArcGis Version 10.2 (ESRI, Redlands, CA, USA) was used to map the study hospital and referring hospitals, using the boundaries of provinces and countries from www.gadm.org.

The baseline demographic and clinical characteristics of the overall cohort are detailed in Table 1. Of 4,989 patients, 2,656 (53%) were male and the median age was 57 (IQR 41–71, range 18–101) years. Two thousand one hundred and ninety nine (44%) patients had at least one comorbidity. The most frequent comorbidities were hypertension (24%), diabetes mellitus (20%) and chronic kidney disease (11%). Of the cohort, 1,173 (24%) were non-transferred patients, 3,524 (71%) were transferred from one of 25 district hospitals, five specialty hospitals, or three private hospitals in Ubon Ratchathani province, and 292 (6%) were transferred from 30 hospitals in other provinces (Fig 2). Of 3,816 transferred patients, 3,245 (85%) and 458 (12%) were transferred within 24 and 48 hours of presentation at the referring hospitals, respectively. Most patients had symptoms for less than eight days prior to admission. Of the cohort, 298 (6%) were admitted directly to an ICU.

**Table 1:** Baseline characteristics of infected patients with and without organ dysfunction within 24 hours of admission.

Parameters	Total Cohort N = 4989	Infection with organ dysfunction^1^ (Sepsis) (n = 3716)	Infection without organ dysfunction^1^ (n = 1273)	P value
**Male gender (n [%])**	**2659 (53%)**	**2139 (58%)**	**520 (41%)**	**<0.001**
**Age (years) (median [IQR])**	**57 (41–71)**	**59 (44–72)**	**51 (34–66)**	**<0.001**
Age group (years) (n [%])				
18–40	1140 (23%)	726 (20%)	414 (33%)	<0.001
>40–60	1543 (31%)	1135 (31%)	408 (32%)	
>60–70	909 (18%)	715 (19%)	194 (15%)	
>70	1397 (28%)	1140 (31%)	257 (20%)	
**Comorbidities(n [%])**				
Hypertension	1190 (24%)	935 (25%)	255 (20%)	<0.001
Diabetes mellitus	1006 (20%)	788 (21%)	218 (17%)	0.002
Chronic kidney disease	545 (11%)	515 (14%)	30 (2%)	<0.001
Dyslipidemia	296 (6%)	211 (6%)	85 (7%)	0.19
Heart disease	282 (6%)	224 (6%)	58 (5%)	0.05
Chronic obstructive pulmonary disease	157 (3%)	126 (3%)	31 (2%)	0.09
Liver disease	133 (3%)	124 (3%)	9 (1%)	<0.001
Cerebrovascular disease	97 (2%)	83 (2%)	14 (1%)	0.01
Malignancy	82 (2%)	57 (2%)	25 (2%)	0.30
Human immunodeficiency virus (HIV)	63 (1%)	37 (1%)	26 (2%)	0.004
**Transferred from other hospitals (n [%])**	3816 (76%)	3246 (87%)	570 (45%)	<0.001
**Duration of symptoms**				
≤ 2 days	2186 (44%)	1660 (45%)	526 (41%)	<0.001
3–7 days	2343 (47%)	1806 (49%)	537 (42%)	
> 7 days	460 (9%)	250 (7%)	210 (17%)	
**Presenting clinical syndromes**^**2**^ **(n [%])**				
Acute febrile illness	1665 (33%)	1123 (30%)	542 (43%)	<0.001
Lower respiratory infection	1454 (29%)	1060 (29%)	394 (31%)	0.10
Diarrheal illness	522 (10%)	411 (11%)	111 (9%)	0.02
Septic shock	1446 (29%)	1416 (38%)	30 (2%)	<0.001
Sepsis	560 (11%)	488 (13%)	72 (6%)	<0.001
Others	700 (14%)	459 (12%)	241 (19%)	<0.001

^**1**^Organ dysfunction is defined by modified SOFA ≥2

^**2**^Patients may have more than one presenting clinical syndrome.

Of 4,989 patients, 3,716 (74%) met criteria for sepsis within the first 24 hours, with a modified SOFA score ≥2. The median of a modified SOFA score among sepsis patients was 5 (IQR 3–7, range 2–21). The most common organ dysfunction observed among sepsis patients was renal dysfunction (68% [2,519/3,716]), followed by cardiovascular (59% [2,174/3,716]), coagulation (50% [1,858/3,716]), liver (30% [1,129 /3,716]), lung (24% [887/3,716]), and central nervous system [CNS] dysfunction (19% [689/3,716]). In general, patients with sepsis were older and were more likely to have common co-morbidities, including hypertension, diabetes mellitus and chronic kidney disease, compared to those without sepsis (Table 1). Sepsis patients were more likely to be transferred from other hospitals and to have shorter duration of symptoms (Table 1).

Of clinical syndromes defined by clinicians, the most frequently diagnosed were acute febrile illness (33%), septic shock (29%), and lower respiratory infections (29%; Table 1). Clinician-defined acute febrile illness and lower respiratory infection were more common in both infected patients with and without sepsis whereas clinician-defined sepsis, and septic shock were more common in patients with organ dysfunction (classified as sepsis in this study). The primary diagnoses of attending physicians included ‘sepsis’ (13%) and ‘septic shock’ (38%) in half (51%) of patients with sepsis defined by a modified SOFA score ≥2 (Table 1).

### Study point-of-care-assessments

Lactate, blood glucose and peripheral capillary oxygen saturation levels were measured at enrollment, and values were available for 4,874 (98%), 4,957 (99%) and 4,915 (98%) patients, respectively. Blood lactate levels were greater than the upper limit of normal (>2.2 mmol/L) in 1,818 (36%) patients. Blood glucose levels were elevated (>140 mg/dL) in 2,035 (41%) patients. Low oxygen saturation (SpO_2_ <90%) was observed in 120 (2%) patients at enrollment. Abnormal GCS (≤14) was observed in 376 patients (8%) at enrollment and more than 40% of these patients had GCS <10.

### Pathogenic organisms isolated within 24-hours of admission

Of 4,989 patients, 752 (15%) had blood culture positive for Gram-negative bacteria (489 [10%]), Gram-positive bacteria (181 [4%]), polymicrobial infections (64 [1%]) and fungi (18 [0.4%]). The most common organism were *Escherichia coli* (209 [4%]), *Burkholderia pseudomallei* (149 [3%]), coagulase-positive staphylococcus (62 [1.2%]), *Klebsiella pneumoniae* (38 [0.8%]), and group A streptococcus (36 [0.7%]) (Table 2).

**Table 2:** Pathogenic organisms from 4,989 patients isolated within 24 hours of admission.

Organisms	Total Cohort N = 4989	Infection with organ dysfunction^1^ (Sepsis) (n = 3716)	Infection without organ dysfunction^1^ (n = 1273)
**Gram negative bacteria**	**489 (9.8%)**	**405 (10.9%)**	**84 (6.6%)**
* Escherichia coli*	209 (4.2%)	165 (4.4%)	44 (3.5%)
* Burkholderia pseudomallei*	149 (3.0%)	131 (3.5%)	18 (1.4%)
* Klebsiella pneumoniae*	38 (0.8%)	33 (0.9%)	5 (0.4%)
* Pseudomonas spp*	34 (0.7%)	26 (0.7%)	8 (0.6%)
* Acinetobacter spp*	32 (0.6%)	27 (0.7%)	5 (0.4%)
* Enterobacter spp*	7 (0.1%)	6 (0.2%)	1 (0.1%)
* Aeromonas spp*	6 (0.1%)	6 (0.2%)	0 (0.0%)
* Proteus spp*	6 (0.1%)	4 (0.1%)	2 (0.2%)
* Salmonella enterica*			
* •* Non-typhoidal *Salmonella*	5 (0.1%)	4 (0.1%)	1 (0.1%)
*• S*. *enterica serotype Typhi*	2 (0.04%)	2 (0.1%)	0 (0.0%)
*Vibrio vulnificus*	1 (0.0%)	1 (0.03%)	0 (0.0%)
**Gram positive bacteria**	**181 (3.6%)**	**143 (3.8%)**	**38 (3.0%)**
Coagulase-positive staphylococcus	62 (1.2%)	46 (1.2%)	16 (1.3%)
Group A streptococcus	36 (0.7%)	31 (0.8%)	5 (0.4%)
Group B streptococcus	31 (0.6%)	23 (0.6%)	8 (0.6%)
Group D streptococcus	9 (0.2%)	8 (0.2%)	1 (0.1%)
* Streptococcus pneumoniae*	17 (0.3%)	13 (0.3%)	4 (0.3%)
* *Other streptococci	14 (0.3%)	11 (0.3%)	3 (0.2%)
* Enterococcus spp*	10 (0.2%)	9 (0.2%)	1 (0.1%)
* *Other Gram positives	2 (0.04%)	2 (0.1%)	0 (0.0%)
**Fungi**	**18 (0.4%)**	**11 (0.3%)**	**7 (0.6%)**
*Cryptococcus neoformans*	6 (0.1%)	3 (0.1%)	3 (0.2%)
*Penicillium marneffei*	6 (0.1%)	3 (0.1%)	3 (0.2%)
*Candida albicans*	2 (0.04%)	1 (0.0%)	1 (0.1%)
Other candida	1 (0.02%)	1 (0.0%)	0 (0.0%)
Unspecified fungi	3 (0.1%)	3 (0.1%)	0 (0.0%)
**Polymicrobial infections**	64 (1.3%)	52 (1.4%)	12 (0.9%)
**Overall**	752 (15.1%)	611 (16.4%)	141 (11.1%)

^**1**^ Organ dysfunction is defined by modified SOFA ≥2.

### Clinical outcomes

**Fig 3:**
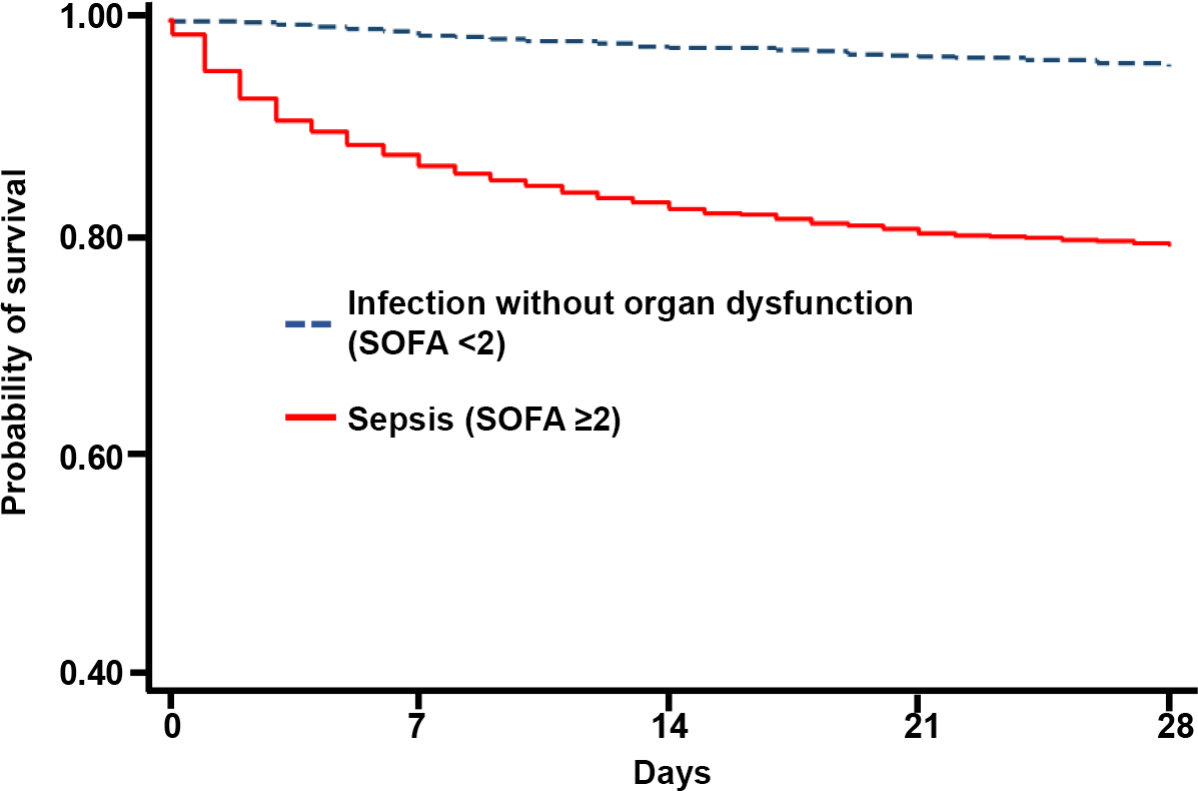
Survival curve comparing infected patients without organ dysfunction to patients with sepsis.

Eight hundred and twenty one deaths occurred in the study period, yielding an overall 28-day mortality of 16% (Table 3). Patients with sepsis had a 28-day mortality of 21% (765/3,716) compared with 4% (54/1,273) in those without sepsis (p<0.001, Fig 3). Among those who died within 28 days, the median time to death was shorter in those with sepsis compared to those without sepsis (5 [IQR 2–11] vs. 12.5 [IQR 7–20], p < 0.001). Among survivors to 28 days (n = 4,170), the length of hospital stay at the study hospital was longer in those with sepsis (4 [IQR 3–7] vs. 3 [IQR 2–6] days, p <0.001). Of 298 patients who were admitted directly to an ICU, 128 (43%) died within 28 days. The median time to death for these ICU patients was 2 days (IQR 1–7 days). Among 170 survivors who were admitted directly to an ICU, the length of hospital stay was 7 days (IQR 4–12 days).

**Table 3:** Outcomes of infected patients with and without organ dysfunction within 24 hours of admission.

Outcomes	Total Cohort N = 4989	Infection with organ dysfunction^1^ (sepsis) (n = 3716)	Infection without organ dysfunction^1^ (n = 1273)	P value
**28-day mortality** **(n [%])**	819 (16%)	765 (21%)	54 (4%)	<0.001
**Time to death (days, median [IQR])** ^**2**^	5 (2–12)	5 (2–11)	13 (7–20)	<0.001
**Length of hospital stay in survivors (days, median [IQR])** ^**3**^	4 (3–7)	4 (3–7)	3 (2–6)	<0.001

^**1**^Organ dysfunction is defined by modified SOFA ≥2.

^**2**^Among those who died within 28 days.

^**3**^Among those who survived to 28 days.

### Factors associated with death

In a univariable Cox proportional hazards model, male gender, older age, transfer from another hospital, higher modified SOFA score, comorbidities and blood culture positive for pathogenic organisms were associated with mortality (S2 Table). Dysfunction in any of the six individual organ systems (respiratory, coagulation, liver, cardiovascular, CNS and renal) was associated with mortality. Nonetheless, the highest risk of mortality was observed in patients with CNS dysfunction (crude hazard ratio [HR] 4.77, 95% CI 4.14–5.49, p<0.001) or with respiratory system dysfunction (crude HR 4.42, 95% CI 3.85–5.08, p<0.001). An association between duration of symptoms and mortality was not observed.

In a multivariable Cox proportional hazards model, transfer from another hospital (adjusted HR 2.18, 95% CI 1.69–2.81), p<0.001) and blood culture positive for pathogenic organisms (adjusted HR 2.21, 95% CI 1.89–2.58), p<0.001) were significantly associated with mortality adjusting for age, gender, sepsis on admission and comorbidities (Table 4). Chronic liver disease and malignancy were significantly associated with mortality, while chronic kidney disease was borderline associated with mortality (Table 4). A sensitivity analysis was conducted by replacing a categorical variable of sepsis on admission (modified SOFA score ≥2) with the continuous modified SOFA score on admission, and transfer from other hospitals was still significantly associated with mortality (adjusted HR 1.61, 95% CI 1.25–2.08, p<0.001).

**Table 4:** Factors associated with 28-day mortality using multivariable Cox proportional hazards model.

Factors	Died (n = 819)	Survived (n = 4170)	Adjusted hazard ratio (95% CI)	P value
**Male gender (n [%])**	473 (58%)	2186 (52%)	1.15 (1.00–1.33)	0.05
**Age group (years) (n [%])**				
18–40	68 (8%)	1072 (26%)	1.0	<0.001
>40–60	235 (29%)	1308 (31%)	1.99 (1.51–2.62)	
>60–70	164 (20%)	745 (18%)	2.17 (1.62–2.90)	
>70	352 (43%)	1045 (25%)	3.25 (2.49–4.24)	
**Transferred from other hospital (n [%])**	749 (91%)	3067 (74%)	2.18 (1.69–2.81)	<0.001
**Infection with organ dysfunction** **(sepsis) within 24 hours of admission (n [%])**	765 (93%)	2951 (71%)	3.30 (2.48–4.41)	<0.001
**Comorbidities (n [%])**				
Diabetes mellitus	213 (26%)	793 (19%)	1.14 (0.97–1.34)	0.12
Chronic kidney disease	142 (17%)	403 (10%)	1.18 (0.98–1.43)	0.09
Liver disease	39 (5%)	94 (2%)	1.78 (1.29–2.47)	0.001
Malignancy	25 (3%)	57 (1%)	2.15 (1.44–3.21)	<0.001
**Blood culture positive for pathogenic organisms**	229 (28%)	523 (13%)	2.21 (1.89–2.58)	<0.001

## Discussion

In this large prospective observational study in a tertiary care hospital in Northeast Thailand, we demonstrated that sepsis is a significant cause of mortality and morbidity in persons presenting with community-acquired infection. Approximately 75% of enrolled patients had evidence of organ dysfunction consistent with sepsis, and 21% of these patients died. Of surviving patients, those with sepsis had longer hospital stays than patients admitted without sepsis, reflecting the added burden of sepsis to patients and to the healthcare system.

Although the study was conducted at a single-center, our screening process evaluated nearly 29,000 patients presenting with suspected infectious diseases over four years. About 13% of the screened patients had evidence of sepsis (3,716/28,752). This proportion is relatively higher than the proportion of sepsis among all hospital admissions in the United States (6%) [30]. The difference could be because our study was screening patients at emergency department, medical wards and medical ICUs, and focusing only on community-acquired sepsis among patients with suspected infection. It is also possible that incidence rates of community-acquired sepsis is more common in LMICs, and further studies are needed. Interestingly, about 75% of enrolled patients were transferred from a multitude of other hospitals in the region. Thus our study cohort captured patients from a large catchment area with a broad distribution of community-acquired infections. Although Thailand is an upper-middle income country, our study highlighted the need to transfer patients with community-acquired infection and sepsis long distances from secondary to tertiary care hospitals. The distance from the referring hospital to the study hospital could be up to 200 kilometers and the travel duration could be up to two to three hours by ambulance. Transferred patients had two-fold higher risk of death after adjusting for age, sex, modified SOFA score, and underlying diseases. This observation is similar to that reported in a recent study in the US observing a positive association between inter-hospital transfer and mortality [32]. While transferred patients are likely to be sicker than those who are not transferred (despite attempts to account for this in our models), additional explanations for this finding could be long distance and time to travel, and limitations of care prior to and during transportation even in resource-rich settings [33]. In our setting, which is similar to other LMICs, diagnostic procedures required by sepsis patients such as microbiology testing and specialized supportive care (including ICUs) are limited in rural hospitals, thus necessitating transfer to tertiary-care hospitals. As the majority of patients are transferred, this finding suggests that developing and implementing strategies to detect and treat sepsis at the referring hospital, responding to transfer requests in a timely fashion, having established transfer protocols in place, and providing appropriate care for sepsis patients during transfer may reduce mortality.

Early recognition of sepsis facilitates early diagnosis and effective management, hence improving outcomes. Based on our data, clinician recognition of sepsis syndromes at the tertiary hospital at the time of admission was moderate. About half of sepsis patients were diagnosed by clinicians as having sepsis or septic shock, and the remaining patients were diagnosed with system-specific illness such as acute febrile illness, lower respiratory infection, diarrheal illness. Recognition of sepsis may be lower in smaller hospitals than in the tertiary care hospital and may hinder the rapid management and supportive care of sepsis prior to transfer, during transfer and on admission in the resource-limited setting. Our observed mortality of community-acquired sepsis patients (21%; N = 3,716) was comparable to those observed in other low and middle income countries, including Haiti (24%; N = 99) [34] and in Thailand, Vietnam and Indonesia (15%; N = 740) [35]. However, the mortality observed is still higher than the mortality of patients with suspected infection and admitted to the ICUs in the recent study in developed countries (16%; N = 7,932) [29].

The common pathogens observed in our study is consistent with the previous findings in Northeast Thailand [36] and in Southeast Asia [35]. *B*. *pseudomallei*, the cause of melioidosis, is also a common cause of community-acquired bacterial infections in Australia [37] and Laos [38]. Our observed proportion of bacteremia was higher than the previous prospective study of community-acquired infections in Southeast Asia (15% vs. 12%) [36], and that could be due to different settings, study areas and study period. Our findings also suggest that community-acquired sepsis at a tertiary-care hospital in LMICs is usually managed outside ICU. Further studies on sepsis management, and its association with mortality outcome are in progress.

Our study has a number of strengths, including the large sample size, diverse catchment area in Northeast Thailand, sequential screening of eligible patients nearly continuously for four years, determination of sepsis within 24 hours of admission, inclusion of medical and ICU wards, and high rates of follow-up for 28-day outcome. There are also several limitations. First, although we conducted detailed assessments, specific tests were not always available or conducted (e.g. arterial blood gas) due to a lack of resources. This lack of testing could lead to an underestimate of the prevalence of sepsis [39, 40]. Second, in addition to unavailability of some data, we did not capture doses of adrenergic agents used. As a consequence, we made slight modifications to the SOFA score to diagnose organ dysfunction and therefore sepsis. This may restrict comparisons of our results to other cohorts characterized by SOFA scores. Third, we did not include patients admitted to surgical or other non-medical wards, who may have different transfer patterns, co-morbidities, management, and outcomes than the medical patients studied. Our follow up was limited to 28 days and does not capture subsequent morbidity or mortality. Fourth, we did not evaluate epidemiology and outcome of sepsis patients who die at referring hospitals or during transfer. Despite these limitations, our study is one of the largest studies to date examining the clinical epidemiology and outcomes of community-acquired infection and sepsis in an LMIC region.

### Conclusions

In our very large prospective observational study of adults with community-acquired infection in Northeastern Thailand, we found that sepsis is associated with a high mortality and morbidity. Transferred patients with infection were at increased risk of death. Limitation of care prior to and during transfer could be the factor associated with mortality. In light of the paucity of data about sepsis in LMICs, this study provides novel information about the significant health impact of this syndrome and underscores the importance of future research to reduce the burden of sepsis.

## Supporting information

S1 Table Systemic manifestation of infection criteria used for screening. (PDF)

S2 Table Factors associated with 28-day mortality using univariable Cox proportional hazards model. (DOCX)
