# Correction: Clinical epidemiology and outcomes of community acquired infection and sepsis among hospitalized patients in a resource limited setting in Northeast Thailand: A prospective observational study (Ubon-sepsis)

**DOI:** 10.1371/journal.pone.0301218

**Published:** 2024-03-21

**Authors:** Viriya Hantrakun, Ranjani Somayaji, Prapit Teparrukkul, Chaiyaporn Boonsri, Kristina Rudd, Nicholas P. J. Day, T. Eoin West, Direk Limmathurotsakul

A classification of patients with sepsis (modified SOFA score > = 2) classification appears incorrectly throughout the article. The incorrect total number of patients with organ dysfunction is 3,716/4,989, and the correct total number is 3,806/4,989.

Blood culture positivity appears incorrectly throughout the article. The incorrect positivity is 752/4,989 (15%) and the correct positivity is 629/4989 (13%).

These changes impact the manuscript, tables, Figs 2 and 3, and the Supporting Information. The authors have provided corrected versions of the tables, figures, and Supporting Information here.

**Fig 2:**
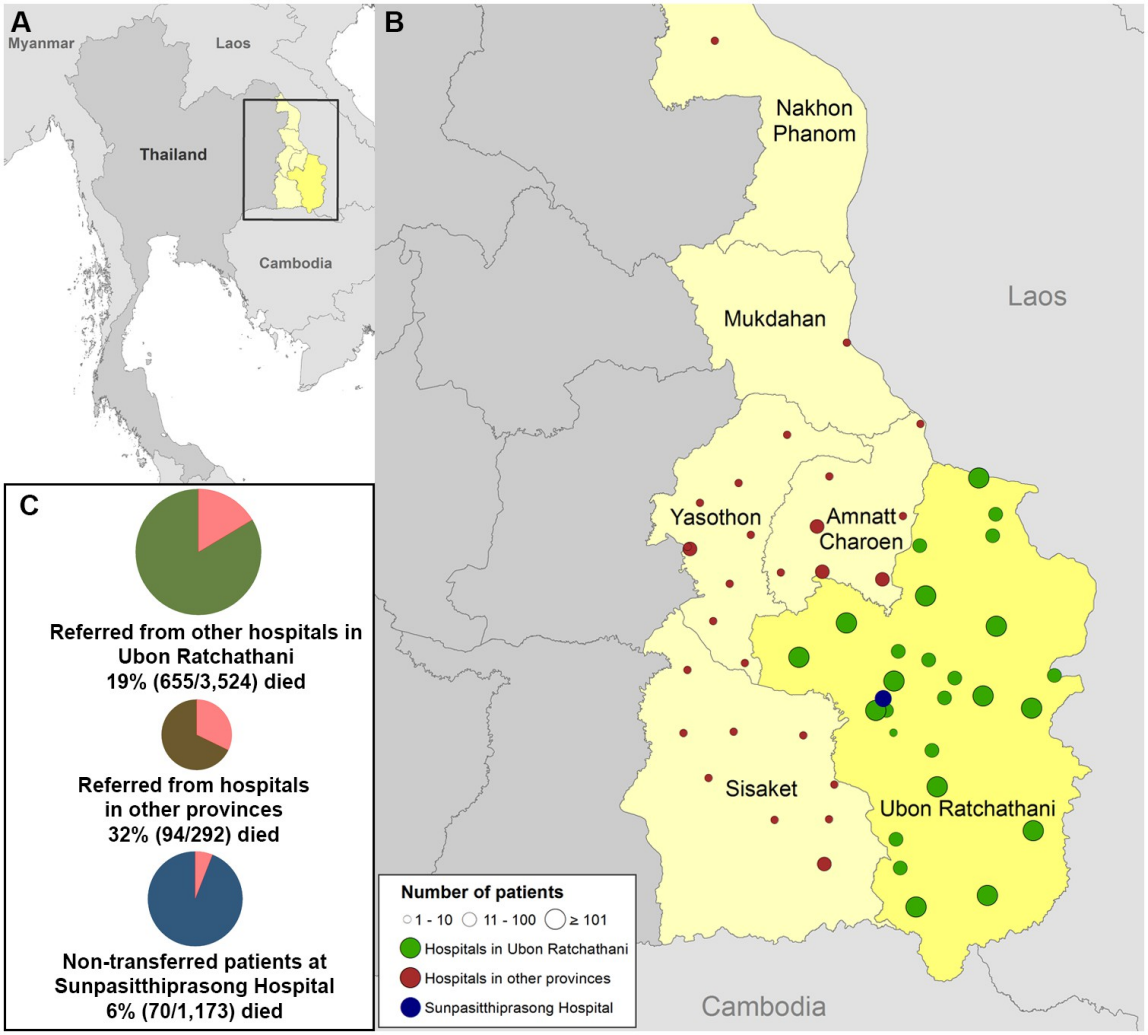
Geographical distribution of the referring hospitals, and 28-day mortality of non-transferred and transferred patients. (A) Map of Thailand. Yellow areas represent provinces from which patients were transferred. (B) Locations of hospitals. Navy blue circle represents the study hospital, Sunpasitthiprasong Hospital. There were a total of 63 referring hospitals; 33 were located in Ubon Ratchathani province, 25 were located in the three adjacent provinces, and 5 were located in the other provinces. Green circles represent 33 referring hospitals located in Ubon Ratchathani province (7 were in Mueang district). Brown circles represent referring hospitals located in three adjacent provinces and the other provinces. (C) Three pie charts represent 28-day mortality. The navy blue, green and brown pie charts represent non-transferred patients, patients transferred from other hospitals in Ubon Ratchathani, and patients transferred from other provinces, respectively. ArcGis Version 10.2 (ESRI, Redlands, CA, USA) was used to map the study hospital and referring hospitals, using the boundaries of provinces and countries from www.gadm.org.

**Fig 3:**
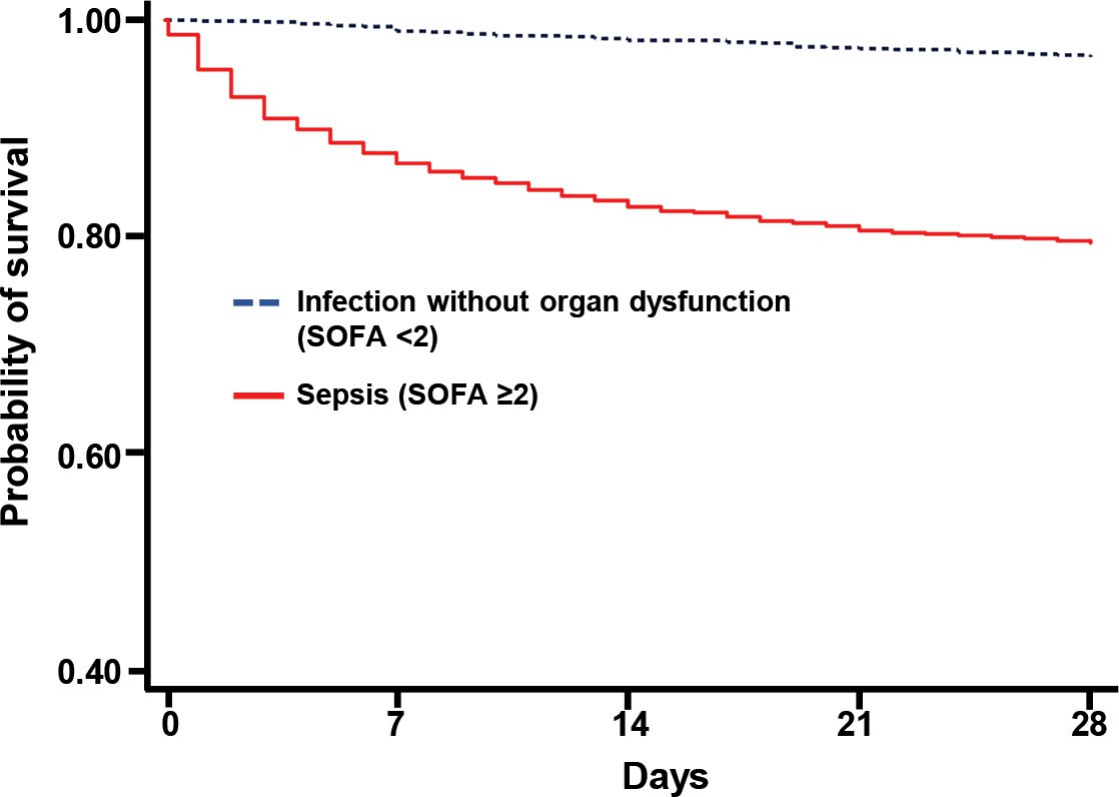
Survival curve comparing infected patients without organ dysfunction to patients with sepsis.

**Table 1:** Baseline characteristics of infected patients with and without organ dysfunction within 24 hours of admission.

Parameters	Total Cohort N = 4989	Infection with organ dysfunction^1^ (Sepsis) (n = 3806)	Infection without organ dysfunction^1^ (n = 1183)	P value
**Male gender (n [%])**	**2659 (53%)**	2179 (57%)	480 (41%)	<0.001
**Age (years) (median [IQR])**	**57 (41–71)**	60 (44–73)	50 (33–65)	<0.001
**Age group (years) (n [%])**				
18–40	1140 (23%)	747 (20%)	393 (33%)	<0.001
>40–60	1543 (31%)	1152 (30%)	391 (33%)	
>60–70	909 (18%)	727 (19%)	182 (15%)	
>70	1397 (28%)	1180 (31%)	217 (18%)	
**Comorbidities(n [%])**				
Hypertension	1190 (24%)	965 (25%)	225 (19%)	<0.001
Diabetes mellitus	1006 (20%)	807 (21%)	199 (17%)	0.001
Chronic kidney disease	545 (11%)	520 (14%)	25 (2%)	<0.001
Dyslipidemia	296 (6%)	218 (6%)	78 (7%)	0.27
Heart disease	282 (6%)	231 (6%)	51 (4%)	0.02
Chronic obstructive pulmonary disease	157 (3%)	133 (3%)	24 (2%)	0.01
Liver disease	133 (3%)	124 (3%)	9 (1%)	<0.001
Cerebrovascular disease	97 (2%)	84 (2%)	13 (1%)	0.02
Malignancy	82 (2%)	60 (2%)	22 (2%)	0.50
Human immunodeficiency virus (HIV)	63 (1%)	39 (1%)	24 (2%)	0.01
**Transferred from other hospitals (n [%])**	3816 (76%)	3310 (87%)	506 (43%)	<0.001
**Duration of symptoms**				
≤ 2 days	2186 (44%)	1696 (45%)	490 (41%)	<0.001
3–7 days	2343 (47%)	1850 (49%)	493 (42%)	
> 7 days	460 (9%)	260 (7%)	200 (17%)	
**Presenting clinical syndromes**^2^ **(n [%])**				
Acute febrile illness	1665 (33%)	1146 (30%)	519 (44%)	<0.001
Lower respiratory infection	1454 (29%)	1113 (29%)	341 (29%)	0.78
Diarrheal illness	522 (10%)	414 (11%)	108 (9%)	0.09
Septic shock	1446 (29%)	1420 (37%)	26 (2%)	<0.001
Sepsis	560 (11%)	498 (13%)	62 (5%)	<0.001
Others	700 (14%)	469 (12%)	231 (20%)	<0.001

^**1**^Organ dysfunction is defined by modified SOFA ≥2,

^**2**^Patients may have more than one presenting clinical syndrome.

**Table 2:** Pathogenic organisms from 4,989 patients isolated within 24 hours of admission.

Organisms	Total Cohort N = 4989	Infection with organ dysfunction^1^ (Sepsis) (n = 3806)	Infection without organ dysfunction^1^ (n = 1183)
**Gram negative bacteria**	430 (8.6%)	370 (10%)	60 (5.1%)
*Escherichia coli*	189 (3.8%)	155 (4.1%)	34 (2.9%)
*Burkholderia pseudomallei*	150 (3.0%)	131 (3.04%)	19 (1.6%)
*Klebsiella pneumoniae*	28 (0.6%)	27 (0.7%)	1 (0.1%)
*Pseudomonas spp*	26 (0.5%)	22 (0.6%)	4 (0.3%)
*Acinetobacter spp*	11 (0.2%)	11 (0.3%)	0 (0.0%)
*Enterobacter spp*	5 (0.1%)	5 (0.1%)	0 (0.0%)
*Aeromonas spp*	6 (0.1%)	6 (0.2%)	0 (0.0%)
*Proteus spp*	4 (0.1%)	3 (0.1%)	1 (0.1%)
*Salmonella enterica*			
• Non-typhoidal *Salmonella*	8 (0.2%)	7 (0.2%)	1 (0.1%)
• *S*. *enterica* serotype Typhi	2 (0.04%)	2 (0.1%)	0 (0.0%)
*Vibrio vulnificus*	1 (0.02%)	1 (0.0%)	0 (0.0%)
**Gram positive bacteria**	152 (3.1%)	116 (3.1%)	36 (3.0%)
Coagulase-positive staphylococcus	53 (1.1%)	37 (1.0%)	16 (1.4%)
Group A streptococcus	26 (0.5%)	23 (0.6%)	3 (0.3%)
Group B streptococcus	30 (0.6%)	21 (0.6%)	9 (0.8%)
Group D streptococcus	7 (0.1%)	6 (0.2%)	1 (0.1%)
*Streptococcus pneumoniae*	17 (0.3%)	13 (0.3%)	4 (0.3%)
Other streptococci	12 (0.2%)	9 (0.2%)	3 (0.3%)
*Enterococcus spp*	6 (0.1%)	6 (0.2%)	0 (0.0%)
Other Gram positives	1 (0.02%)	1 (0.03%)	0 (0.0%)
**Fungi**	22 (0.4%)	15 (0.4%)	7 (0.6%)
*Cryptococcus neoformans*	7 (0.1%)	4 (0.1%)	3 (0.2%)
*Penicillium marneffei*	6 (0.1%)	3 (0.1%)	3 (0.2%)
*Candida albicans*	3 (0.1%)	2 (0.1%)	1 (0.1%)
*Candida parapsilosis*	1 (0.02%)	1 (0.03%)	0 (0.0%)
Other candida	2 (0.04%)	2 (0.1%)	0 (0.0%)
Unspecified fungi	2 (0.04%)	2 (0.1%)	0 (0.0%)
*Fusarium spp*	1 (0.02%)	1 (0.03%)	0 (0.0%)
**Polymicrobial infections**	25 (0.5%)	21 (0.6%)	4 (0.3%)
**Overall**	629 (12.6%)	522 (13.7%)	107 (9.0%)

^**1**^ Organ dysfunction is defined by modified SOFA ≥2.

**Table 3:** Outcomes of infected patients with and without organ dysfunction within 24 hours of admission.

Outcomes	Total Cohort N = 4989	Infection with organ dysfunction^1^ (sepsis) (n = 3806)	Infection without organ dysfunction^1^ (n = 1183)	P value
**28-day mortality (n [%])**	819 (16%)	779 (20%)	40 (3%)	<0.001
**Time to death (days, median [IQR])** ^2^	5 (2–12)	5 (2–11)	14(7–21)	<0.001
**Length of hospital stay in survivors (days, median [IQR])** ^3^	4 (3–7)	4 (3–7)	3 (2–6)	<0.001

^**1**^Organ dysfunction is defined by modified SOFA ≥2

^**2**^Among those who died within 28 days

^**3**^Among those who survived to 28 days

**Table 4:** Factors associated with 28-day mortality using multivariable Cox proportional hazards model.

Factors	Died (n = 819)	Survived (n = 4170)	Adjusted hazard ratio (95% CI)	P value
**Male gender(n [%])**	473 (58%)	2186 (52%)	1.13 (0.99–1.31)	0.08
**Age group (years) (n [%])**				
18–40	68 (8%)	1072 (26%)	1.0	<0.001
>40–60	235 (29%)	1308 (31%)	2.01 (1.53–2.64)	
>60–70	164 (20%)	745 (18%)	2.18 (1.63–2.92)	
>70	352 (43%)	1045 (25%)	3.24 (2.49–4.23)	
**Transferred from other hospital (n [%])**	749 (91%)	3067 (74%)	2.08 (1.61–2.68)	<0.001
**Infection with organ dysfunction (sepsis) within 24 hours of admission (n [%])**	765 (93%)	2951 (71%)	4.08 (2.93–5.67)	<0.001
**Comorbidities (n [%])**				
Diabetes mellitus	213 (26%)	793 (19%)	1.12 (0.95–1.32)	0.18
Chronic kidney disease	142 (17%)	403 (10%)	1.19 (0.98–1.44)	0.07
Liver disease	39 (5%)	94 (2%)	1.77 (1.28–2.45)	0.001
Malignancy	25 (3%)	57 (1%)	2.10 (1.41–3.14)	<0.001
**Blood culture positive for pathogenic organisms**	196 (24%)	433 (10%)	2.18 (1.85–2.56)	<0.001

## Supporting information

S1 Table Systemic manifestation of infection criteria used for screening. (DOCX)

S2 Table Factors associated with 28-day mortality using univariable Cox proportional hazards model. (DOCX)
