# Supplementary material for: Clinical epidemiology and outcomes of community acquired infection and sepsis among hospitalized patients in a resource limited setting in Northeast Thailand: A prospective observational study (Ubon-sepsis)
Source: PLoS One. 2018 Sep 26;13(9):e0204509. doi: 10.1371/journal.pone.0204509 (PMC6157894; doi:10.1371/journal.pone.0204509)
Supplement: S1 Table — (PDF) [file pone.0204509.s001.pdf]

S1 Table. Systemic manifestation of infection criteria used for screening

General parameters

1. Fever or hypothermia (Core body temperature defined as > 38.3C or < 36.0C)
2. Tachycardia (heart rate > 90 beats per minute)
3. Tachypnea (respiratory rate > 20 per minute)
4. Altered mental status with Glasgow Coma Score (GCS) < 15 or <10 if intubated
5. Hyperglycemia (plasma glucose > 140 mg/dL) in the absence of diabetes

Inflammatory parameters

6. Leukocytosis (white blood cell count > 12,000/µL), leukopenia (white blood cell count < 4000/µL) or immature forms > 10%
7. Plasma C-reactive protein >2 SD above the normal value
8. Plasma procalcitonin >2 SD above the normal value

Hemodynamic parameters

9. Arterial hypotension (systolic blood pressure (SBP) < 90 mmHg, mean arterial pressure (MAP) < 70 mmHg, or SBP decrease > 40 mmHg)

Organ dysfunction parameters

10. Low oxygen saturation determined by pulse oximetry (SpO_2 <95%) determined by pulse oximetry
11. Arterial hypoxemia (PaO_2 / FIO_2 < 300)
12. Acute oliguria (urine output < 0.5 mL/kg/hr or 45 mmol/L for 2 hours)
13. Creatinine increase > 0.5 mg/dL
14. Coagulation abnormalities (international normalised ratio >1·5 or activated partial thromplastin time >60 seconds)
15. Thrombocytopenia (Platelet count < cells 100,000/µL)
16. Ileus (absent bowel sounds)
17. Hyperbilirubinaemia (plasma total bilirubin > 4 mg/dL)

Tissue perfusion parameters

18. Hyperlactatemia (> 1 mmol/L)
19. Decreased capillary refill or mottling
20. Significant edema or positive fluid balance
