# Supplementary material for: Clinical epidemiology and outcomes of community acquired infection and sepsis among hospitalized patients in a resource limited setting in Northeast Thailand: A prospective observational study (Ubon-sepsis)
Source: PLoS One. 2018 Sep 26;13(9):e0204509. doi: 10.1371/journal.pone.0204509 (PMC6157894; doi:10.1371/journal.pone.0204509)
Supplement: S2 Table — (DOCX) [file pone.0204509.s002.docx]

**S2 Table. Factors associated with 28-day mortality using univariable Cox proportional hazards model**

| **Factors** | **Died**  **(n=819)** | **Survived**  **(n=4170)** | **Crude hazard ratio**  **(95% CI)** | **p value** |
| --- | --- | --- | --- | --- |
| **Male gender, n (%)** | 473 (58%) | 2186 (52%) | 1.21 (1.06- 1.39) | 0.01 |
| **Age group (years), n (%)** |  |  |  |  |
| 18-40 | 68 (8%) | 1072 (26%) | 1.0 | <0.001 |
| >40-60 | 235 (29%) | 1308 (31%) | 2.70 (2.06- 3.53) |  |
| >60-70 | 164 (20%) | 745 (18%) | 3.21 (2.42- 4.27) |  |
| >70 | 352 (43%) | 1045 (25%) | 4.63 (3.57- 6.00) |  |
| **Comorbidities, n (%)** |  |  |  |  |
| Hypertension | 236 (29%) | 954 (23%) | 1.32 (1.13- 1.53) | <0.001 |
| Diabetes mellitus | 213 (26%) | 793 (19%) | 1.44 (1.23- 1.68) | <0.001 |
| Chronic kidney disease | 142 (17%) | 403 (10%) | 1.81 (1.51- 2.17) | <0.001 |
| Dyslipidemia | 51 (6%) | 245 (6%) | 1.05 (0.79- 1.39) | 0.74 |
| Heart disease | 54 (7%) | 228 (5%) | 1.20 (0.91- 1.57) | 0.21 |
| Chronic obstructive pulmonary disease | 39 (5%) | 118 (3%) | 1.58 (1.14- 2.18) | 0.005 |
| Liver disease | 39 (5%) | 94 (2%) | 2.00 (1.45- 2.76) | <0.001 |
| Cerebrovascular disease | 23 (3%) | 74 (2%) | 1.52 (1.00- 2.30) | 0.05 |
| Malignancy | 25 (3%) | 57 (1%) | 1.98 (1.33- 2.95) | 0.001 |
| Human immunodeficiency virus (HIV) | 7 (1%) | 56 (1%) | 0.65 (0.31- 1.36) | 0.25 |
| **Transfer from other hospitals, n (%)** | 749 (91%) | 3067 (74%) | 3.52 (2.76- 4.50) | <0.001 |
| **Duration of symptoms (days), median (IQR)** | 3 (1-5) | 3 (1-5) | 1.00 (0.99- 1.01) | 0.67 |
| **Infection with organ dysfunction  (sepsis), n (%)** | 765 (93%) | 2951 (71%) | 5.35 (4.06- 7.05) | <0.001 |
| **Organ system dysfunction, n (%)** |  |  |  |  |
| Respiration | 362 (44%) | 526 (13%) | 4.42 (3.85- 5.08) | <0.001 |
| Coagulation | 381 (47%) | 1581 (38%) | 1.40 (1.22- 1.60) | <0.001 |
| Liver | 291 (36%) | 873 (21%) | 1.98 (1.71- 2.28) | <0.001 |
| Cardiovascular | 510 (62%) | 1789 (43%) | 2.09 (1.81- 2.40) | <0.001 |
| Central nervous system | 318 (39%) | 399 (10%) | 4.77 (4.14- 5.49) | <0.001 |
| Renal | 622 (76%) | 2129 (51%) | 2.81 (2.39- 3.30) | <0.001 |
| **Total modified SOFA score, median (IQR)** | 6 (4-9) | 3 (1-5) | 1.25 (1.23- 1.27) | <0.001 |
| **Blood culture positive for pathogenic organisms, n (%)** | 229 (28%) | 523 (13%) | 2.46 (2.11- 2.86) | <0.001 |
